# Synthetic data generation with probabilistic Bayesian Networks

**DOI:** 10.3934/mbe.2021426

**Published:** 2021-10-09

**Authors:** Grigoriy Gogoshin, Sergio Branciamore, Andrei S. Rodin

**Affiliations:** Department of Computational and Quantitative Medicine, Beckman Research Institute, and Diabetes and Metabolism Research Institute, City of Hope National Medical Center, 1500 East Duarte Road, Duarte, CA 91010 USA

**Keywords:** Bayesian networks, synthetic data generation, Directed Acyclic Graph, probabilistic graphical models, Markov blanket, central limit

## Abstract

Bayesian Network (BN) modeling is a prominent and increasingly popular computational systems biology method. It aims to construct network graphs from the large heterogeneous biological datasets that reflect the underlying biological relationships. Currently, a variety of strategies exist for evaluating BN methodology performance, ranging from utilizing artificial benchmark datasets and models, to specialized biological benchmark datasets, to simulation studies that generate synthetic data from predefined network models. The last is arguably the most comprehensive approach; however, existing implementations often rely on explicit and implicit assumptions that may be unrealistic in a typical biological data analysis scenario, or are poorly equipped for automated arbitrary model generation. In this study, we develop a purely probabilistic simulation framework that addresses the demands of statistically sound simulations studies in an unbiased fashion. Additionally, we expand on our current understanding of the theoretical notions of causality and dependence / conditional independence in BNs and the Markov Blankets within.

## 1. Introduction

Dependency modeling is a descriptive / predictive modeling activity that is especially suited to a systems biology approach to data analysis (in particular, analysis of heterogeneous big data). During dependency modeling, a biological network, or a graphical representation of a biological model (including genotypes, phenotypes, metabolites, endpoints, and other variables and their interrelationships), is constructed from the ”flat” data. (Subsequently, specific components of biological networks can be evaluated using conventional statistical criteria, and sub-networks can be selected for further biological hypothesis generation and testing). This activity is also known as causal discovery (inference) in graphical models. Bayesian Networks (BNs)-based dependency modeling is an established computational biology tool that is rapidly gaining acceptance in the diverse areas of biomedical data analysis, ranging from molecular evolution [1] to chromatin interactions [2] to flow cytometry [3] to diagnostic and prognostic clinical applications [4, 5] to genomics [6–9] to transcriptomics [10–12] to metabolomics [13] to signaling [14] to microarray data analysis [15] to general multi-omics [16–19]. Comprehensive treatments of BN methodology can be found in textbooks [20–23] and review papers [24–27].

Most of the recent work in the BN methodology field aims at improving the scalability and accuracy of BN modeling [28–31], handling mixed data types (including various types of biological data) [32–34], incorporating latent variables [35, 36], causal discovery [26, 27, 37], and developing more robust software interfaces and visualization options [38]. In view of these developments, the ability to objectively assess the performance of BN modeling is of the utmost importance. Currently, there are three principal avenues for accomplishing this: (i) using well-established (in the machine learning community) predefined benchmark models and datasets, such as “Alarm” or “Asia”, (ii) using various specialized biologically-oriented benchmark datasets, both real and simulated, such as ”LUCAS” or “DREAM” [9, 30, 39, 40], and (iii) developing approximately realistic simulation frameworks [31, 32, 41, 42]. The first approach is necessarily limited and does not generalize to modern high-dimensional biological data. The latter two strategies have their pros and cons (primarily having to do with observability - generalizability trade offs); a useful discussion can be found in [9].

In this study, we concentrate on the third approach, namely constructing robust, generalizable, and mathematically rigorous simulation frameworks. Typically, these frameworks involve (i) specifying synthetic network structures, (ii) utilizing some interaction model to encode this structure, and (iii) the actual generation of synthetic data via a method that depends on the interaction model. The interaction model is highly contextual, but the most common approaches are either additive noise modeling [43], e.g. SEM (structural equation modeling) [31, 41], or probabilistic modeling via specification of the distribution [5, 44, 45]. The latter requires sampling techniques to generate data, e.g. exact sampling, MCMC or Gibbs sampling.

In the broader context of heterogeneous biological data and networks, it is important to distinguish between the notions of causality, correlation, association and dependence. Establishing causality [21,24,27,40] is usually perceived as the ultimate goal in the biomedical network analyses [16,29,31]. However, establishing (and even defining) causality is often equivocal, for both theoretical [24, 27] and practical/biological reasons (e.g., the ambiguity of the “causal” interpretation of the relationship between two correlated gene expression measurements, or two single nucleotide polymorphisms in linkage disequilibrium [41]). Of course, there are advantages to viewing certain biological network models (or their parts) through the lens of directional causality. First, directional causality often corresponds well to biological reality (to use an obvious example, phenotypes tend to be ”downstream” of genotypes). Second, existing BN algorithmic machinery largely relies on the concept of directed acyclic graphs (DAGs). And yet, at the most basic level (e.g., physical - chemical interaction of two biological molecules), it is unclear if it is possible, or even desirable, to impose a “directional causation” label on what is essentially a dependence relationship that is inferred from observing the joint probability distribution. The concept of ”cause - effect” may have more to do with rationalization in seeking implication chains rather than reflecting the totality of any given interaction. Therefore, we chose to frame this study predominantly around the concept of dependence, which is a measure of relevance that allows us to make inferences about biological reality without necessarily invoking considerations of causality (however, we do take into consideration ancestor-offspring relationships in DAGs).

Regardless of whether we focus on causation or dependence relationships, SEM, being informationally denser and, therefore, a less general model of interaction [43], might not be the most appropriate tool for generating synthetic data in the case of biological BNs. Leaving aside the issues of acyclicity (and directionality in general), and the assumptions of linearity and normality (which can be dealt with, if imperfectly), there is a fundamental issue of observability. Indeed, consider a typical biological dataset subject to BN modeling — “real” biological datasets include only comparatively small sets of variables that are observable under the confines of specific biomedical studies (i.e., experiments). These variables cannot always be expected to be in any particular categorizable relationship known or suspected a priori. The most one can postulate with certainty about a ”real” dataset is that the variables in it may be dependent or independent. Even for the well-dissected biological systems, we cannot necessarily say that the captured variables, no matter how carefully selected, belong to a group of key components that adequately describe the structure and dynamics of a biological system in question. Consequently, we propose to use a fully probabilistic, unconstrained, framework for synthetic data generation purposes, equipped for the statistical study of model parameter space as well as for the statistical performance assessment of structure discovery methods.

It is built around an analytically defined distribution induced by a given graphical model and, coupled with random model structure generation, is immediately interpretable as the joint probability distribution, while simultaneously featuring inferential characteristics encoded in conditional independencies. Below we detail considerations involved in building such a framework in Methods, Proofs and Results: Sections 2–3; ascertain the congruence between the ”forward” process (simulations) and ”inverse” process (actual reconstruction from the data of the networks and Markov Blankets within) in Methods, Proofs and Results: Section 4; and discuss simulation sample size considerations in Results: Section 5.

## 2. Methods, proofs and results: synthetic data generation with probabilistic networks

Synthetic data generation is the so-called *forward problem* in which data is obtained from the model under consideration given a set of known parameters. Similarly, the so-called *inverse problem* is concerned with identifying model parameters that fit the data, which, in our case, constitutes reconstruction of a BN from the observations with an assumption that the observations correspond to a probabilistic model.

Here, we are predominantly interested in identifying the challenges arising in the context of statistical verification, validation, and quality assessment of BN reconstruction algorithms. From this perspective, the data synthesis algorithm should be able to generate samples from arbitrarily diverse models to cover as much of a model space as possible.

The analytically defined distribution induced by a given BN model, has a joint probability that is factorizable into an expression that is no more complex than the methodical application of the chain rule permits. However, the set of simplifications imposed by conditional independencies encoded in the BN may significantly lower the complexity of the factorization.

Hence, the joint probability distribution of a given model can be represented as a product of conditional and unconditional probabilities

(2.1) $$P\left(\underset{i}{\cap }X_{i}\right)=\underset{i}{\prod }P\left(X_{i}|\underset{j>i}{\cap }X_{j}\right)$$

This product provides a way to generate sample components serially from local conditional probabilities instead of direct sampling from the n-dimensional joint distribution. This is an important computational consideration, because using direct sampling to obtain adequate representation for low probability events is difficult given a finite sample size.

In fact, this difficulty grows with the dimensionality of the model, so that as the number of variables increases, many joint events quickly become intractable even for binary variables. Therefore, being able to rely on local sampling of conditional probability distributions is a key factor in successfully synthesizing components of an adequate n-dimensional sample that actually reflects the fine structure of the joint distribution.

If a necessary level of resolution in the data sampled from a given joint distribution is not achieved, the data source uniqueness problem, typical for BN reconstruction setting, is likely to be amplified. That is, if the same data is obtainable from different source distributions, making the source distinction all but impossible, the problem of recovering a BN from such data will be more ill-posed.

In order to utilize the resolution provided by the expansion above, a strategy for methodically generating conditional distributions for the dependent variables needs to be specified:

For *n*-variate variable *X* and *m*-variate variable *Y* the relationship between the two variables can be characterized via the law of total probability which, combined with the chain rule, reveals the role of conditional probability:

(2.2) $$P(X)=\overset{m}{\underset{i=1}{\sum }}P\left(X\cap Y=y_{i}\right)=\overset{m}{\underset{i=1}{\sum }}P\left(X|Y=y_{i}\right)P\left(Y=y_{i}\right)$$

where *y*_*i*_ are individual disjoint events.

This marginalization procedure can be viewed as a matrix-vector product which defines the conditional probability *P*(*X*|*Y*) as an *n* × *m* matrix with entries *P*(*X* = *x*_*j*_|*Y* = *y*_*i*_):

(2.3) P(X)=P(X∣Y)⋅P(Y)

where *P*(*X*) = (*P*(*x*_0_), …, *P*(*x*_*n*_))^*T*^, *P*(*Y*) = (*P*(*y*_0_), …, *P*(*y*_*m*_))^*T*^ with the subscripted values representing individual disjoint events.

Note the fraction *P*(*X* = *x*_*j*_ ∩ *Y* = *y*_*i*_)/*P*(*Y* = *y*_*i*_) arising from the use of the chain rule cannot be used to define conditional probability, because this expression is not defined for zero probability events of *Y*.

A formal criterion for the *conditional independence* of *X* and *Y* is given by

(2.4) $$\forall y_{i}\;\left(P\left(X|Y=y_{i}\right)=P(X)\right)$$

Its negation provides the criterion for the *conditional dependence*

(2.5) $$\exists y_{i}\;\left(P\left(X|Y=y_{i}\right)\neq P(X)\right)$$

i.e. the existence of at least one dependent event *y*_*i*_ is sufficient to establish the dependence.

The above condition, however, reveals little about how to consistently construct appropriate objects, and how the properties of these objects reflect on the character of the dependence relationship between *X* and *Y*. This can be remedied by assessing the dependence relationship in a way that is agnostic about the unconditional distributions, as a separately defined transformation of the ancestor distribution ***q*** = *P*(*Y*) to the descendant distribution ***p*** = *P*(*X*), a matrix *P*(*X*|*Y*) relating *X* to *Y* via marginalization operation, inducing *P*(*X* ∩ *Y*) and *P*(*X*) along the way, i.e.

(2.6) $$q\overset{P(X|Y)}{\to }p$$

With the above goal in mind, consider the following observation

**Lemma 1.** *Let X be an n-variate random variable, then the columns of conditional distribution P*(*X*|*Y*) *are members of* (*n* − 1)*-simplex* $S\subset {\mathbb{R}}^{n}$.

*Proof.* Trivially, the elements of any column of a conditional distribution *P*(*X*|*Y*) sum up to 1. Hence, because the variable *X* has *n* outcomes, all columns are members of the set defined by

(2.7) $$S=\left\{\left(v_{1},\ldots ,v_{n}\right):\overset{n}{\underset{i=1}{\sum }}v_{i}=1,\;v_{i}\geq 0,\;\forall i\right\}$$

which is an *n* − 1-simplex, and a subset of ${\mathbb{R}}^{n}$ □

To simplify the notation and to emphasize that the conditional distribution *P*(*X*|*Y*) is defined independently, let *D* be an *n* × *m* linear operator whose columns are members of the (*n* − 1)-simplex *S*, so that *P*(*X*) = *D* · *P*(*Y*) given some *P*(*Y*).

**Lemma 2.** *If the column rank of D defined above is at least two, then X defined by P*(*X*) = *D* · *P*(*Y*) *is conditionally dependent on Y.*

*Proof.* Suppose the rank of *D* is at least two, then there is at least one linearly independent column, i.e.

(2.8) $$\exists i,\;\forall \alpha_{k}\geq 0,\;\left(D_{i}\neq \underset{k\neq i}{\sum }D_{k}\alpha_{k}\right)$$

where *D*_*i*_ is the *i*-th column of *D*. It follows that if for every index *k* ≠ *i* the coefficients are given by $\alpha_{k}=\frac{P\left(y_{k}\right)}{1-P\left(y_{i}\right)}$, then

(2.9) $$D_{i}\left(1-P\left(y_{i}\right)\right)\neq \underset{k\neq i}{\sum }D_{k}P\left(y_{k}\right)$$

Rearranging the terms yields

(2.10) $$\exists i,\;\left(D_{i}\neq D\cdot P(Y)\right)$$

In other words, there is an *i* corresponding to *y*_*i*_ such that *P*(*X*|*Y* = *y*_*i*_) ≠ *P*(*X*), which is a restatement of the dependence criterion. Hence, there is at least one dependent event, making *X* dependent on *Y*. □

This lemma already guarantees that any matrix of appropriate size with rank of at least two should be sufficient to generate a conditionally dependent pair of variables. However, this does not elucidate additional constraints that an arbitrary conditional dependence may impose, and it does little to provide control over the structure of a synthetically constructed dependence relationship.

The following lemma addresses these shortcomings:

**Lemma 3.** *Let X be an n-variate random variable dependent on an m-variate random variable Y; then*

- the convex hull C formed by the columns of D has at least two vertices;
- rank of D must be at least two;
- P(X) lies in the convex hull C formed by the columns of D;
- the number of vertices of convex hull C corresponds to the number of conditionally dependent events.

*Proof.* Suppose *X* is conditionally dependent on *Y*, then

(2.11) $$\exists y_{i},\;\left(D_{i}\neq D\cdot P(Y)\right)$$

where *D*_*i*_ is the *i*-th column of *D*, because *P*(*X*) = *D* · *P*(*Y*). Rearranging the terms gives

(2.12) $$D_{i}\left(1-P\left(Y=y_{i}\right)\right)\neq \underset{k\neq i}{\sum }D_{k}P\left(Y=y_{k}\right)$$

for some arbitrary *P*(*Y*). Assuming that *P*(*Y* = *y*_*i*_) ≠ 1, this yields

(2.13) $$D_{i}\neq \underset{k\neq i}{\sum }D_{k}\alpha_{k}$$

with coefficients defined on the probability simplex characterized by

(2.14) $$\underset{k\neq i}{\sum }\alpha_{k}=1,\;\alpha_{k}\geq 0,\forall k\neq i$$

In other words, for *X* to be conditionally dependent on *Y* for any *D* defined independently of *P*(*Y*), there must be at least one column in *D* not expressible as a convex combination of other columns. Hence, this column is a vertex of a convex hull *C* ⊂ *S* formed by all convex combinations of columns of *D* characterized by

(2.15) $$\overset{m}{\underset{i=1}{\sum }}D_{i}\alpha_{i},\;\overset{m}{\underset{i=1}{\sum }}\alpha_{i}=1,\;\alpha_{i}\geq 0,\forall i$$

The above implies the following:

- the convex hull contains more than one member, and, therefore, has at least two vertices;
- because there are at least two vertices, this polytope must be embedded in at least a (2–1)-simplex, and, therefore, in at least ${\mathbb{R}}^{2}$ - hence the linear operator *D* must be at least of rank two;
- the unconditional distribution *P*(*X*) always lies in this convex hull, because *P*(*X*) = *D* · *P*(*Y*) for any independently defined *D* and *P*(*Y*);
- every conditionally dependent event of *Y* corresponds to a vertex of the convex hull that envelops the columns of *D*. □

Some of the immediately useful consequences of the above can be summarized in the following corollary:

**Corollary 1.** *Let X be an n-variate random variable, Y be an m-variate random variable and D a linear operator characterized by P*(*X*) = *D* · *P*(*Y*)*, then*

- X is conditionally dependent on Y if and only if the rank of the linear operator D is at least two.
- Let d be the number of dependent ancestor events that the linear operator D of the size n × m and rank r encodes; then r ≤ d ≤ m.

Conditional distributions of higher ancestral complexity, i.e. *P*(*X*|∩_*k*_*Y*_*k*_), can be treated as a pairwise distribution *P*(*X*|*Y*) if we observe that ∩_*k*_*Y*_*k*_ can be viewed as a single variable over the combinatorically prescribed joint events. In this situation, the only specification that is different from the already considered scenario with the pairwise conditional distribution is that the joint events are not given *a priori*. Rather, the joint events have to be derived as a function of the number and arity of the ancestor variables in the DAG. For example, for two binary ancestor variables, there will be four possible events, six for a binary and a tertiary variable, etc.

After defining all the necessary parameters, the sample construction can be carried out via the exact sampling method, starting with the sampling of the set of root nodes, and proceeding sequentially forward following the rule that, given a particular ancestral sample y*, the joint probability distribution for the downstream node combined with the ancestral sample is given by

(2.16) $$P\left(X\cap Y=y^{*}\right)=P\left(X|Y=y^{*}\right)P\left(Y=y^{*}\right)$$

i.e. for each sample *Y* = y* the sample *X* = x* is obtained with probability *P*(*X* = x*|*Y* = y*). This serialized construction of samples will produce a single row of data with values corresponding to nodes in the probabilistic DAG. This row will then constitute a single sample from the given DAG. Repeating this procedure as necessary will generate a dataset of a prescribed size. We discuss sample size considerations below in Results: Section 5.

The above results suggest an explicit approach for constructing random conditionally dependent distributions (and generating synthetic data from them) in a formally sound way, while ensuring that all relevant relationship properties are accounted for. This framework is, in principle, extensible to mixed-type variable models for known distribution families via approximations, but arbitrary model generation in that scenario is highly non-trivial due to difficulties with the specification for arbitrary distriubtion families.

It should be noted that the numerical structure of the linear operator *D* is secondary to the properties of the convex hull formed by its columns, because the latter reflects the qualitative structure of the event space of the conditional dependence relationship. In particular, manipulating the properties of this convex polytope provides a way to choose the proportion of dependent and independent events in the event space.

More importantly, we observe that the columns of *D* must be sampled from the (*n* − 1)-simplex *S*, and if sampled uniformly, this is equivalent to sampling from a Dirichlet distribution **Dir**(*α*) with the appropriate concentration parameters. However, as will become evident below, constructing random dependence relationships that adequately cover the model parameter space is not as trivial a task as it may seem, and is something that uniform sampling of individual simplex elements is not able to address.

**Proposition 1.** *Let* ***t***, ***p***, ***q*** *be i.i.d. random variables obtained from* ***Dir***(***α***) *with* ***α*** = (1, 1)*, and let D be a* 2 × 2 *matrix with columns* ***p*** *and* ***q****. Then the distribution of* ***z*** = *D* · ***t*** *is non-uniform.*

*Proof.* Note that all the variables including ***z*** are members of (2 − 1)-simplex, so all variables have two components, i.e. ***z*** = (*z*, 1 − *z*), ***t*** = (*t*, 1 − *t*), ***p*** = (*p*, 1 − *p*) and ***q*** = (*q*, 1 − *q*). Then ***z*** = *D* · ***t*** reduces to the single independent equation relating the components, namely *z* = *tp*+(1 − *t*)*q*. Using the substitution $u=\frac{z-tp}{1-t}$ to reparametrize the integral yields the following expression for the cumulative distribution

(2.17) $$P(tp+(1-t)q\leq z)=\int \int \int_{0}^{(z-tp)\frac{1}{1-t}}f_{t}(t,1-t)f_{p}(p,1-q)f_{q}(q,1-q)dq\;dp\;dt\;=\int_{0}^{z}\int \int f_{t}(t,1-t)f_{p}(p,1-p)f_{q}\left(\frac{u-tp}{1-t},1-\frac{u-tp}{1-t}\right)dp\;dt\frac{du}{1-t}$$

The corresponding density is obtained by differentiating with respect to *z* and evaluating the resulting integrals with the appropriate integration bounds. Using the requirement that $0\leq \frac{z-tp}{1-t}\leq 1$ allows us to infer the integration set for *p* as

(2.18) $$\Omega =\left\{p\;:\text{ max}\left(0,\frac{z-1}{t}+1\right)\leq p\leq \text{min}\left(1,\frac{z}{t}\right)\right\}$$

Applying the above to the evaluation of the integral yields

(2.19) $$f(z)=\int \frac{1}{1-t}\int f_{t}(t,1-t)f_{p}(p,p-1)f_{q}\left(\frac{z-tp}{1-t},1-\frac{z-tp}{1-t}\right)dp\;dt\;=\int_{\delta }^{1}\frac{1}{1-t}\int_{\Omega }dp\;dt\;=\int_{\delta }^{1}\frac{1}{1-t}\left(\text{min}\left(1,\frac{z}{t}\right)-\text{max}\left(0,\frac{z-1}{t}+1\right)\right)dt\;=2(-z\text{ log}(z)-(1-z)\text{log}(1-z))=2\text{H}(z)$$

where **H** is the information-theoretic entropy. Hence, the resulting density is non-uniform. □

The above implies that the naively constructed *D* induces a non-uniform distribution, strongly favoring simplex elements with higher entropy. Furthermore, as the number of columns in *D* grows, ***z*** = *D* · ***t***, viewed as a weighted sum of the columns of *D*, has an asymptotic tendency towards the normal distribution. The latter point is made evident by the following central limit theorem:

**Proposition 2.** *Let D*_*j*_*, a member of m* − 1*-simplex, the i-th column of a m*×*n matrix D, and **t**, a member of n* − 1*-simplex, be random variables obtained from* ***Dir***(***α***) *with* ***α*** = (1, …, 1)*, an m-tuple and an n-tuple respectively. Then each of the components of* ***z*** = *D* · ***t*** *tends towards a normal distribution.*

*Proof.* The components *d*_*ij*_ of *D*_*j*_ are *β*(1, *m* − 1) distributed random variables with common mean and variance

(2.20) $$E\left(d_{ij}\right)=\frac{1}{m},\;\sigma^{2}\left(d_{ij}\right)=\frac{m-1}{m^{2}(m+1)},$$

while the components *t*_*j*_ of ***t*** are *β*(1, *n* − 1) distriubted random variables with common mean and variance

(2.21) $$E\left(t_{j}\right)=\frac{1}{n},\;\sigma^{2}\left(t_{j}\right)=\frac{n-1}{n^{2}(n+1)},$$

Hence, the components *z*_*i*_ of ***z*** are randomly weighted sums

(2.22) $$z_{i}=\overset{n}{\underset{j=1}{\sum }}d_{ij}t_{j}$$

It follows from Theorem 3.1 in [46] that

(2.23) $$\sqrt{n}\left(z_{i}-\mu \right)\overset{d}{\to }\text{N}\left(0,2\sigma^{2}\right)$$

where *μ* = ***E***(*d*_*ij*_) and *σ*^2^ = *σ*^2^(*t*_*j*_). □

The implication of the above is that BN modeling simulations that construct their conditional probabilities *D* in a naive way cannot adequately cover model parameter space for simulation studies as the individual node parameter distributions downstream of the root node in the network will tend towards the maximum entropy states. This tendency may be further amplified down the ancestor/offspring chains, and since for a non-root node, *n* grows exponentially with the number of ancestors, we can expect this bias to be noticeable for practically every non-root node.

One way to rectify this is, for example, by appropriately varying concentration parameters ***α***, associated with conditional probabilities. Another option may be to fix the unconditional distributions of all the nodes of a BN first (thus losing the ability to generate a BN serially), and attempting to obtain all the conditional distributions subject to the constraints imposed by the prescribed unconditional node distributions (possibly too computationally expensive for the setting where millions of BNs and datasets will have to be generated for a sufficiently robust simulation study).

On the other hand, this distributional phenomenon could be instrumental in identifying more realistic prior distributions for constructing scoring criteria. For instance, for the BD (Bayesian-Dirichlet) family of metrics, the prior directly depends on the choice of the concentration parameter ***α***.

Finally, under certain assumptions about the nature of the conditional dependence relationship in a given model, the tendency of the descendant nodes towards higher entropy configuration can be also be used as a criterion for suggesting a partial topological order of variables in the underlying DAG for the purposes of BN reconstruction. That is, reasoning that variables with relatively low entropy are unlikely to be terminal nodes can substantially narrow the space of possible DAG configurations that could fit a given dataset.

## 3. Methods, proofs and results: random graph distribution

To estimate the error of the structure recovery for the inverse solver (i.e., the actual BN reconstruction algorithm), the forward solver (the synthetic data generator) needs to not only synthesize data from a given model, but also generate the random model structures (joint probability factorizations) that the data is sampled from. This can be realized by producing random DAG adjacency matrices, or, more specifically, triangular extractions from symmetric adjacency matrices to account for the acyclicity of structures.

In order for the error estimation to lead to reliable and meaningful results, synthesized random structures should be distributed in a well understood or prescribed manner. Therefore, we need to establish some of the basic properties of DAG structure distributions.

The discrete nature of graph configurations implies nonuniform distribution across various graph densities for combinatorical reasons. For a graph of *n* variables with a given topological ordering, the maximum density (maximum number of edges) possible is given by

(3.1) $$D=\frac{n(n-1)}{2}$$

Then the total count *C* of possible graph configurations with a density *d* < *D* is given by the binomial coefficient

(3.2) $$C(d)=\frac{D!}{d!(D-d)!}$$

which implies that structures with density around $\frac{1}{2}D$ are more numerous than symmetrically higher or lower density structures. The total number of possible configurations of any density is

(3.3) $$\overset{D}{\underset{k=0}{\sum }}C(k)=2^{D}$$

Let *δ* be the density valued function over graph structures. Then the probability that a graph G has density δ(G)=d is

(3.4) $$P(\delta (G)=d)=\frac{C(d)}{2^{D}}$$

The joint probability of obtaining a configuration G with density *d* is therefore

(3.5) $$P(G\cap (\delta (G)=d))=P(G|\delta (G)=d)P(\delta (G)=d)=\frac{1}{C(d)}\frac{C(d)}{2^{D}}=\frac{1}{2^{D}}$$

where the conditional probability of G given the prescribed density is

(3.6) $$P(G|\delta (G)=d)=\frac{1}{C(d)}$$

Of particular interest is the expression for P(δ(G)=d) because it implies that structures of medium density are more likely than any other. This effect reflects another systematic bias in the model space and can severely hinder the performance of a naive stochastic structure recovery method that searches the model space by evaluation only, not accounting for the fact that medium density states will occur more frequently. The same, of course, applies to sampling of the model space for statistical simulation studies.

In principle, this could be remedied by a forced uniform sampling across various density groups, thereby increasing the sampling rate over lower and higher density groups relative to the medium density groups or, similarly, by decreasing the sampling rate over the medium density groups. However, this may be impractical, because even for a problem of moderate size, the number of possible configurations at the extremes of the distribution is sharply limited relative to the medium density graphs, which would make it virtually impossible to maintain a constant sampling error. For a 16-node structure the above translates to 120 configurations with one edge, 840261910995 configurations with 8 edges, and on the order of 10^34^ medium-density configurations with 60 edges. Therefore, if a 10% sampling rate is to be maintained at any cost, then the number of samples across all density groups is in the order of 10^35^ (with most of the computational load in the medium-density groups). We conclude that pursuing a uniform sampling is computationally impractical for all but the smallest models, and, in general, the problem can only be addressed by introducing additional considerations and background information.

## 4. Methods, proofs and results: forward inference over Markov Blankets

It is important to ensure the congruence of independence and probabilistic inference mechanisms in the simulation framework (as described above) and during the BN construction process. In this section, we will dissect the ”localized” probabilistic inference within a BN by utilizing the concept of Markov Blanket.

Consider the fact that in a simple BN of the form *A* → *X* → *D* the state of the variable *X* is not completely determined by the state of its ancestor *A* given that it also has a descendant *D*. The reason is that the conditional probability distribution of *X* for the particular instantiation *a* of the ancestor is further constrained by an instantiation *d* of the descendant, i.e.

(4.1) P(D=d)=P(D=d∣X)⋅P(X)=P(D=d∣X)⋅P(X∣A=a)P(A=a)

The situation quickly gets more complicated for larger BNs, but, fortunately, a complete specification of a variable requires only its Markov Blanket, conveniently factoring the graph.

By definition, a Markov Blanket *M* of a particular variable *X* renders such a ”center” variable conditionally independent from the rest of the BN (i.e. BN sans the Markov Blanket), with the ”periphery” variables of *M* completely determining the state of the center variable assuming their states are known. This naturally leads to the problem of estimating the conditional probability of *X* as a function of the states of periphery variables in *M* − *X*.

(4.2) $$P(X|M-X)=\frac{P(M)}{P(M-X)}$$

The computational difficulties here are the same as with the estimation of any sufficiently complex joint probability. Often, we would lose accuracy due to the sampling error before we can estimate most joint events, unless all the variables are defined analytically, and the dimensionality of the computation for such an estimation grows superexponentially with the number of variables. However, these difficulties can be mitigated by relying on the structural information encoded in the BN. If correct, the simplifications introduced into the factorization sufficiently reduce the computational burden and the sampling error, thereby making the desired estimation not only feasible, but also more reliable. And although some of the difficulties alleviated are traded for the multiplicative propagation of error, the end result is still preferable to the direct loss of resolution.

Let *a*(*X*) and *d*(*X*) denote the set of immediate ancestors and immediate descendants of *X* respectively. Also, let

(4.3) ad(X)=a(d(X))−X−a(X)−d(X)

so that *ad*(*X*) represents the set of ancestors of members of *d*(*X*) excluding *X* itself that are neither direct ancestors or descendants of *X* themselves, i.e.

(4.4) X∉ad(X), ad(X)∩a(X)=∅, ad(X)∩d(X)=∅

Then the Markov Blanket *M* of *X* is

(4.5) M=X∪a(X)∪d(X)∪ad(X)

and the joint probability of M can be factored as follows

(4.6) $$P(M)=P(X,a(X),d(X),ad(X))=P(a(X))P(X|a(X))\underset{Y_{i}\in d(X)}{\prod }P\left(Y_{i}|a\left(Y_{i}\right)\right)\underset{Z_{j}\in ad(X)}{\prod }P\left(Z_{j}|a\left(Z_{j}\right)\right)$$

Note that

(4.7) ad(X)∩d(X)=∅⇒P(ad(X)∣X,a(X))=P(ad(X)∣a(X))

and that

(4.8) a(d(X))∩d(X)≠∅⇒∃Y∈d(X)(a(Y)∩d(X)≠∅)

Equation (4.6) provides direct access to all joint probabilities for any instantiation of variables in *M*, allowing us to proceed with the estimation of the desired conditional probability

(4.9) $$P\left(X=x_{i}|M-X\right)=\frac{P\left(X=x_{i},M-X\right)}{\sum_{j}P\left(X=x_{j},M-X\right)}$$

marginalizing over *X* in the denominator for any given state *x*_*i*_ of *X* in the numerator. Undoubtedly, the division operation itself introduces further numerical error into the estimate, but the classical numerical techniques are applicable in this situation.

Different types of inference are possible under the same assumptions, utilizing the same set of equations. For example, one could assess the probability of a periphery variable *Z* ∈ *M* being in a state *z*_*i*_ in order for the center variable *X* ∈ *M* to be observed in a state *x*_*j*_:

(4.10) $$P\left(Z=z_{i}|X=x_{j},M-X-Z\right)=\frac{P\left(X=x_{j},Z=z_{i},M-X-Z\right)}{\sum_{k}P\left(X=x_{j},Z=z_{k},M-X-Z\right)}$$

In essence, the above type of inquiry allows us to make a judgment about the most likely scenario that coincides with the *X* = *x*_*j*_ event, and this inquiry could be extended to more than one periphery variable.

Furthermore, the idea that periphery variables can predict the state of the center variable with a prescribed accuracy can also be utilized in structural optimization during the BN structure search. Local prediction accuracy as an objective optimization criterion is inherently practical because it attempts to maximize the utility of the result rather than concentrating exclusively on a set of more abstract notions associated with information-theoretic properties.

## 5. Results: effects of the central limit and sample size considerations

We first consider the tendency of terminal node distributions to concentrate in the region of the simplex corresponding to maximum entropy when the nodes of a BN are initialized with conditional probability distributions sampled from the naively configured Dirichlet distribution with unitary concentration parameters, as described in Section 2.

**Figure 1.**
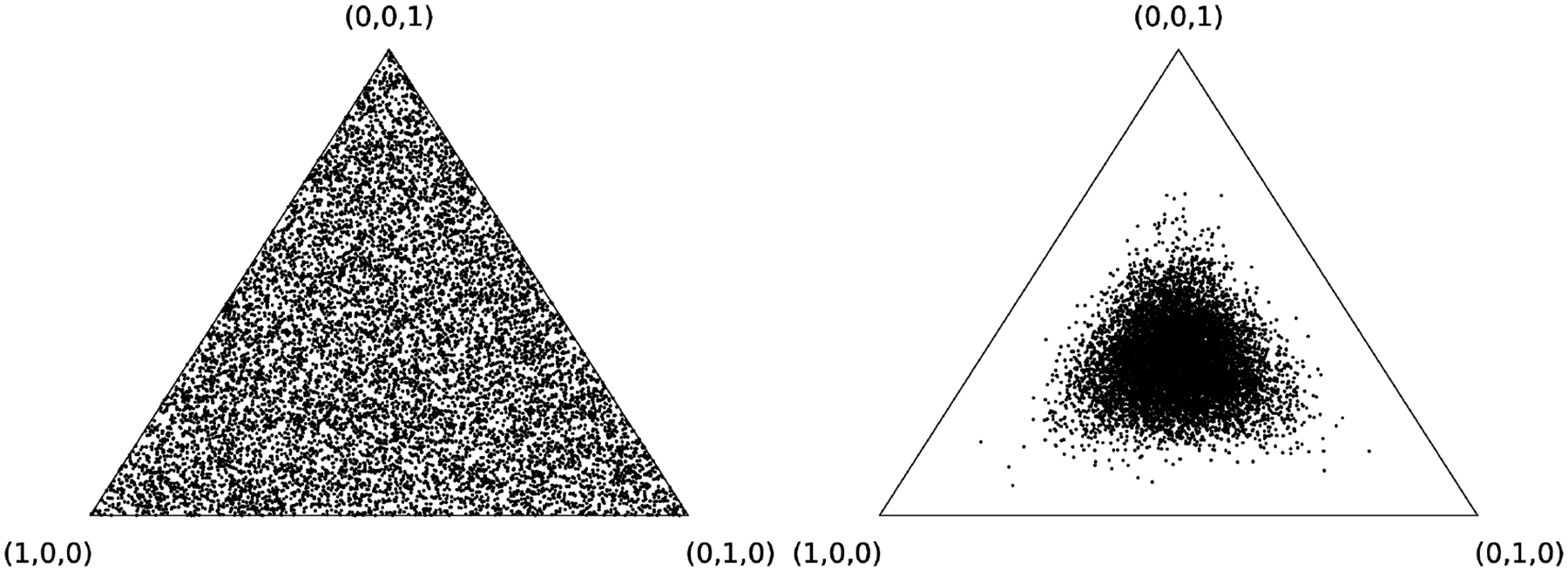
The scatter plot of 10^4^ samples of root node (simplex on the left) and terminal node (simplex on the right) distributions of the randomly generated 8-node BN with tertiary variables. For the simplex on the left the root node distributions are drawn from the Dirichlet distribution with *α* = (1, 1, 1), while the simplex on the right displays the effect of convergence to the central limit on the terminal node distributions.

To simulate this behavior we construct a random BN structure with eight tertiary nodes and prescribed random conditional distributions with columns drawn from the ***Dir***(***α***) with ***α*** = **1**, initialize the root nodes with unconditional probabilities drawn from the same distribution and propagate marginalization consecutively from the root nodes towards the terminal nodes, thus obtaining unconditional probabilities for the whole network (see Figure 1).

As expected, the result is that the terminal nodes tend toward maximum entropy configuration (the central region of the associated simplex on the right, Figure 1) — a manifestation of the central limit condition restricting the admissible node parameters. Under these circumstances, models favoring lower entropy distributions (outer regions of the associated simplex) on the terminal nodes are practically inaccessible and would be unusually rare.

**Figure 2.**
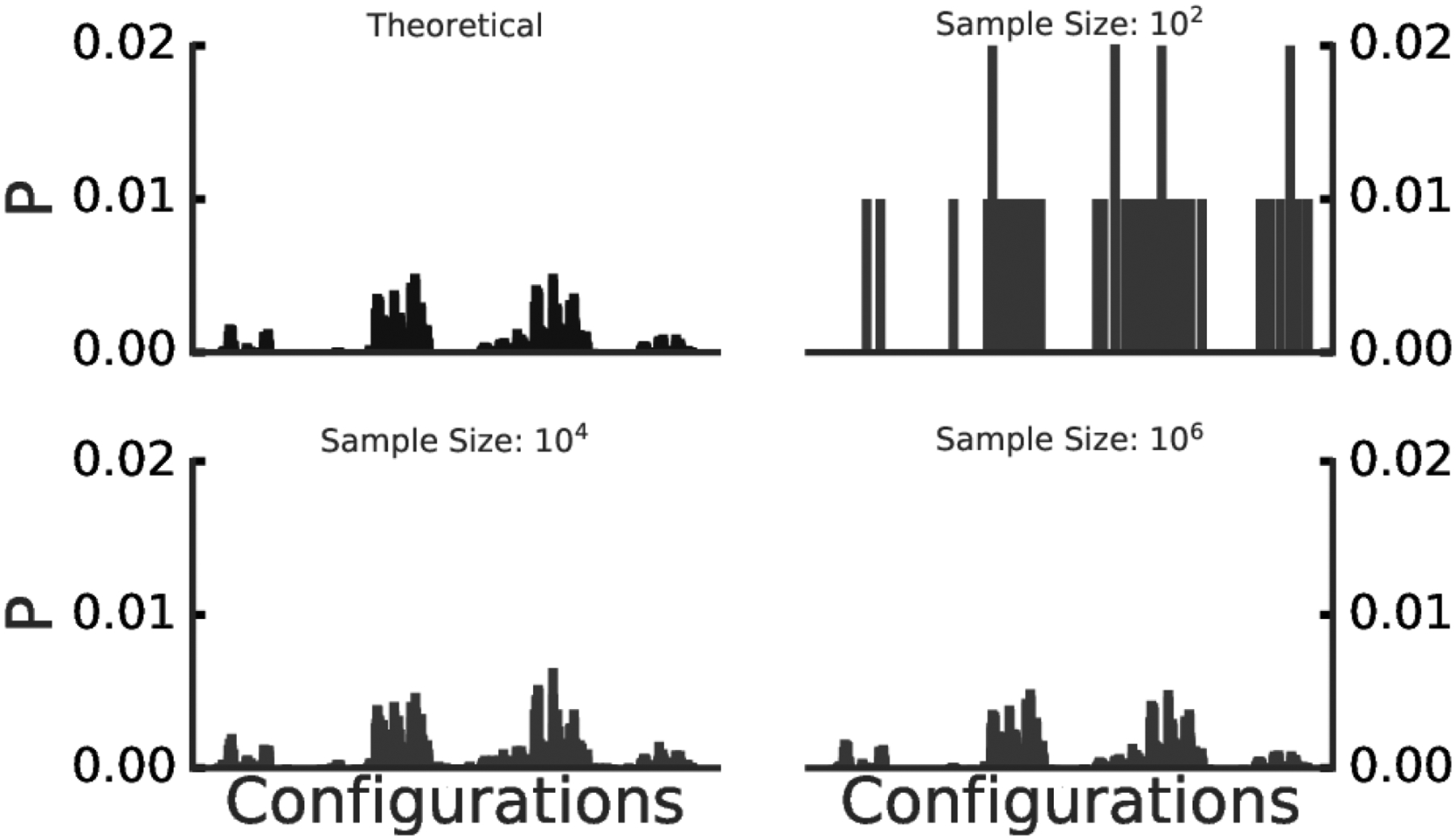
Comparison of the analytical and estimated sampled distributions of all joint events of a given 8-nodes random graph with average variable connection density and arity being 0.8 and 3, respectively. The analytical distribution is shown in the top left panel; distributions estimated using sample size (10^2^, 10^4^, 10^6^) are shown in the top right, bottom left and bottom right panels, respectively.

**Figure 3.**
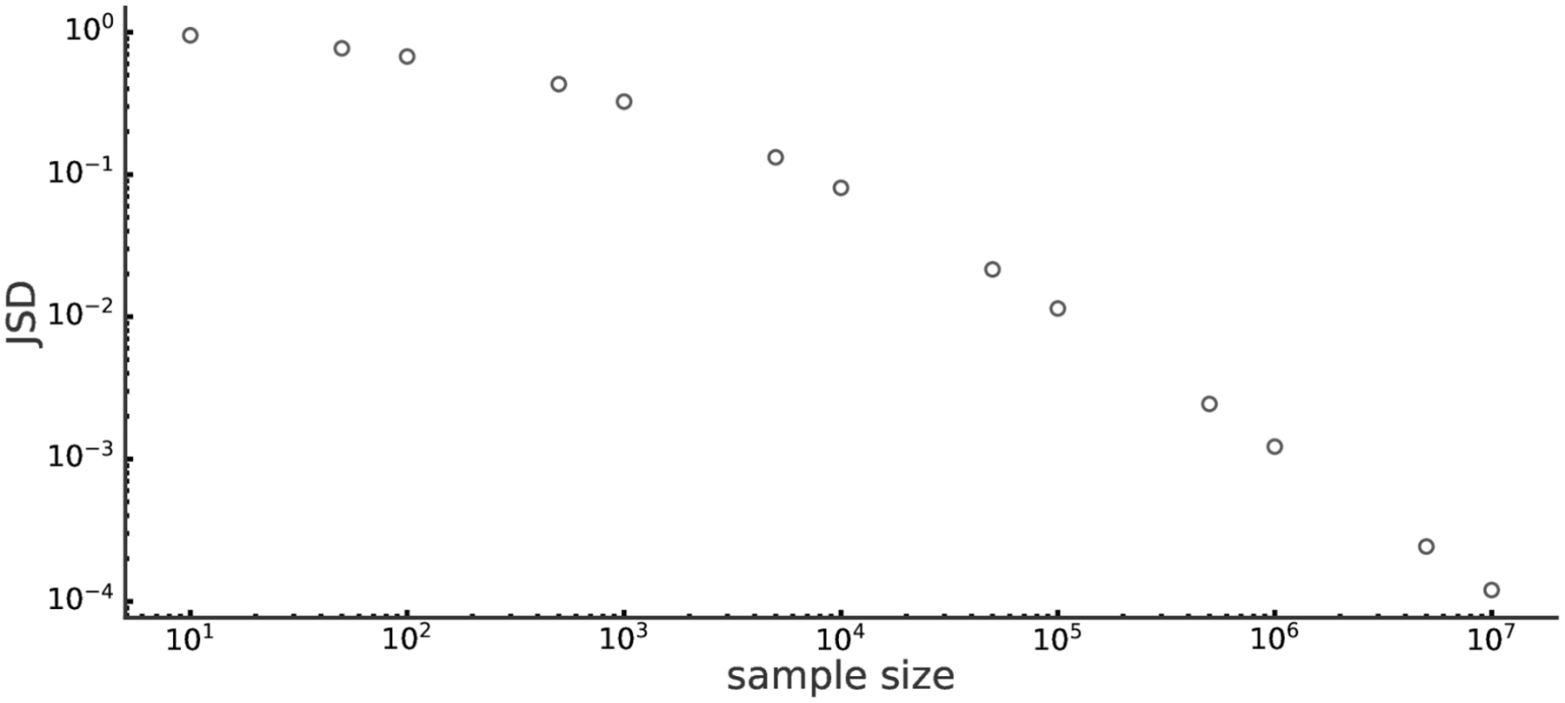
Jensen-Shannon divergence (JSD) between the analytical and estimated sampled distributions of all joint events of a given 8-nodes random graph with average variable connection density and arity being 0.8 and 3, respectively. JSD, shown as a function of sample size, is obtained by averaging 100 replicas for each sample size value.

In practice, it is important to evaluate how many samples should be generated (as prescribed in Section 2) for the estimation of the joint probability to be sufficiently robust. As a representative example, let us consider a network of eight tertiary variables and an average edge density of 0.8. This network has at the most 3^8^ = 6561 possible joint events, and it is straightforward to analytically compute their associated probabilities, provided the network has been fully defined. Subsequently, we can evaluate the effect of sample size in estimating the ”true” distribution. Figure 2 shows the analytical and estimated distribution over all possible joint events in our example, for four (increasing) sample size values. To quantify the differences between the distributions, we used Jensen-Shannon divergence (JSD), a distributional divergence metric (Figure 3). These results confirm that our procedures converge to zero distributional divergence with the increasing sample size.

Although our simulation framework is not limited by the complexity of the model, and the above results generalize to any number of nodes, the 8-node configuration was specifically chosen for the sake of a clearer presentation and for the ability to easily generate the sample size that covers the event space more exhaustively. As can be seen in Figure 3, the sample count required for even a modestly sized network to be well-represented in the data can be substantial.

Given that the analytical joint distribution over the network is calculated during the simulation, the sample size necessary to achieve the desired representational accuracy can be estimated via the multinomial distribution. If ***p*** = (*p*_1_, …, *p*_*k*_) is the density of the joint events, and ***x*** = (*x*_1_, …, *x*_*k*_) are the sample counts of the observed joint events, then the probability of sampling a particular sequence of counts ***x*** is given by the multinomial *f*(***x***, ***p***). This allows us to formulate a number of possible accuracy constraints. For example, controlling the maximum deviation from the idealized count *N****p***, with *N* = ∑_*i*_ *x*_*i*_, can be achieved by estimating N necessary to bring the probability of such a maximum deviation below a certain threshold *ϵ*, i.e. *P*(max_*i*_ |*Np*_*i*_ − *x*_*i*_| > *δ*) < *ϵ*, so that for a defined accuracy level *δ*, all deviations beyond that have a small probability of occurrence.

## 6. Discussion and conclusions

Simulation studies based on synthetic data generation are ubiquitous in the machine learning and computational biology domains. They are instrumental in objectively assessing the performance of modeling methods, such as BNs. However, comprehensive and realistic simulation frameworks are comparatively underdeveloped in the context of network-centered systems biology methodology. In this study, we presented theoretical and methodological considerations for developing a realistic, fully probabilistic, synthetic data generation framework for biological BNs.

At this time, we have developed corresponding algorithms and software for generating synthetic data for randomly generated discrete-variables probabilistic DAGs. These are included as part of our ”BNOmics” BN modeling software package [6], which is freely available from the authors, as well as from the bitbucket open source distributary (https://bitbucket.org/77D/bnomics). In the future, we intend to expand our algorithms to take into account mixed data types (continuous-discrete variable mix, specifically).

Two outstanding issues, relevant to both the BN reconstruction and synthetic data generation / BN performance evaluation, are (i) whether the Markov Blankets (within the BNs) can in principle be recovered with consistency (provided that the “forward generation” and “backward reconstruction” processes are sufficiently aligned, as in our proposed framework), and (ii) whether it is possible to incorporate a strict, rigorous definition of dependence and conditional independence in the BN reconstruction process, as opposed to concentrating on establishing causation from correlation. We are cautiously optimistic on both counts, but more investigation is needed, and will be forthcoming.

Much of the work detailed in this communication was spurred by our ongoing collaborative immuno-oncology study that involves comparative BN analyses of multi-dimensional FACS (fluorescence-activated cell sorting) and other immuno-oncology datasets obtained from patients with gastrointestinal and breast cancers undergoing checkpoint blockade immunotherapy treatments [3]. In that study, we aim to construct and compare BNs representing immune network states in sickness and health, before and after therapy, in responders and non-responders. In the future, we plan to generate synthetic datasets that more closely align with the real, observed FACS and other immuno-oncology datasets — this will allow us to rigorously validate the reconstructed immune network BNs and corresponding biological results. Based on our experience working with such data, it is our opinion that each and every BN analysis should, ideally, be accompanied by a corresponding simulation study built around synthetic data that hews as closely as realistically possible to the actual biological data under consideration.

## Data Availability

Relevant code and software are available directly from the authors, or as a part of the BNOmics package, at https://bitbucket.org/77D/bnomics.

## References

[1] Branciamore S, Gogoshin G, Di Giulio M, Rodin AS, Intrinsic properties of TRNA molecules as deciphered via bayesian network and distribution divergence analysis, Life (Basel), 8 (2018), E5. 29419741 10.3390/life8010005 PMC5871937

[2] Zhang X, Branciamore S, Gogoshin G, Rodin AS, Analysis of high-resolution 3d intrachromosomal interactions aided by bayesian network modeling, Proc. Natl. Acad. Sci. USA, 114 (2017), E10359–E10368. 29133398 10.1073/pnas.1620425114 PMC5715735

[3] Rodin AS, Gogoshin G, Hilliard S, Wang L, Egelston C, Rockne RC, , Dissecting response to cancer immunotherapy by applying bayesian network analysis to flow cytometry data, Int. J. Mol. Sci, 22 (2021), 2316. 33652558 10.3390/ijms22052316 PMC7956201

[4] Sedgewick AJ, Buschur K, Shi I, Ramsey JD, Raghu VK, Manatakis DV, , Mixed graphical models for integrative causal analysis with application to chronic lung disease diagnosis and prognosis, Bioinformatics, 35 (2019), 1204–1212. 30192904 10.1093/bioinformatics/bty769 PMC6449754

[5] Becker AK, Dörr M, Felix SB, Frost F, Grabe HJ, Lerch MM, , From heterogeneous healthcare data to disease-specific biomarker networks: A hierarchical bayesian network approach, PLoS Comput. Biol, 17 (2021). 10.1371/journal.pcbi.1008735 PMC7906470 33577591

[6] Gogoshin G, Boerwinkle E, Rodin AS, New algorithm and software (bnomics) for inferring and visualizing bayesian networks from heterogeneous “big” biological and genetic data, J. Comput. Biol, 24 (2017), 340–356. 27681505 10.1089/cmb.2016.0100 PMC5372779

[7] Rodin A, Brown A, Clark AG, Sing CF, Boerwinkle E, Mining genetic epidemiology data with bayesian networks: Application to apoe gene variants and plasma lipid levels, J. Comput. Biol, 12 (2005), 1–11. 15725730 10.1089/cmb.2005.12.1 PMC1201451

[8] Sherif FF, Zayed N, Fakhr M, Discovering alzheimer genetic biomarkers using bayesian networks, Adv. Bioinform, 2015 (2015), 639367. 10.1155/2015/639367 PMC4561111 26366461

[9] Wang L, Audenaert P, Michoel T, High-dimensional bayesian network inference from systems genetics data using genetic node ordering, Front. Genet, 10 (2019), 1196. 31921278 10.3389/fgene.2019.01196 PMC6933017

[10] Lan Z, Zhao Y, Kang J, Yu T, Bayesian network feature finder (banff): an r package for gene network feature selection, Bioinformatics, 32 (2016), 3685–3687. 27503223 10.1093/bioinformatics/btw522 PMC5181536

[11] Neapolitan R, Xue D, Jiang X, Modeling the altered expression levels of genes on signaling pathways in tumors as causal bayesian networks, Cancer Inform, 13 (2014), 77–84. 10.4137/CIN.S13578 PMC4051800 24932098

[12] van de Stolpe A, Verhaegh W, Blay J-Y, Ma CX, Pauwels P, Pegram M, , RNA based approaches to profile oncogenic pathways from low quantity samples to drive precision oncology strategies, Front. Genet, 11 (2021). 10.3389/fgene.2020.598118 PMC7893109 33613616

[13] Qi Q, Li J, Cheng J, Reconstruction of metabolic pathways by combining probabilistic graphical model-based and knowledge-based methods, BMC Proc, 8 (2014), S5. 10.1186/1753-6561-8-S6-S5 PMC4202177 25374614

[14] Pe’er D, Bayesian network analysis of signaling networks: a primer, Sci. Signal, 2005 (2005), pl4. 10.1126/stke.2812005pl4 15855409

[15] Piatetsky-Shapiro G, Tamayo P, Microarray data mining: facing the challenges, SIGKDD Explor. Newsl, 5 (2003), 1–5.

[16] Zeng Z, Jiang X, Neapolitan R, Discovering causal interactions using bayesian network scoring and information gain, BMC Bioinform, 17 (2016), 221. 10.1186/s12859-016-1084-8 PMC4880828 27230078

[17] Ziebarth JD, Bhattacharya A, Cui Y, Bayesian network webserver: a comprehensive tool for biological network modeling, Bioinformatics, 29 (2013), 2801–3. 23969134 10.1093/bioinformatics/btt472

[18] Zhang Q, Shi X, A mixture copula bayesian network model for multimodal genomic data, Cancer Inform, 16 (2017). 10.1177/1176935117702389 PMC5397279 28469391

[19] Zhao Y, Chang C, Hannum M, Lee J, Shen R, Bayesian network-driven clustering analysis with feature selection for high-dimensional multi-modal molecular data, Sci. Rep, 11 (2021). 10.1038/s41598-021-84514-0 PMC7933297 33664338

[20] Pearl J, Probabilistic reasoning in intelligent systems, 1988.

[21] Pearl J, Causality, Cambridge Univ. Press, 2009.

[22] Russell S, Norvig P, Artificial intelligence: A modern approach, 3rd edition, Prentice Hall, 2010.

[23] Spirtes P, Glymour C, Scheines R, Causation, prediction, and search, 2nd edition, MIT Press, 2000.

[24] Glymour C, Zhang K, Spirtes P, Review of causal discovery methods based on graphical models, Front. Genet, 10 (2019), 524. 31214249 10.3389/fgene.2019.00524 PMC6558187

[25] Heckerman D, Geiger D, Chickering D, Learning bayesian networks: The combination of knowledge and statistical data, Mach. Learn, 20 (1995), 197–243.

[26] Spirtes P, Zhang K, Causal discovery and inference: concepts and recent methodological advances, Appl. Inform. (Berl), 3 (2016), 3. 27195202 10.1186/s40535-016-0018-x PMC4841209

[27] Zhang K, Schölkopf B, Spirtes P, Glymour C, Learning causality and causality-related learning: some recent progress, Natl. Sci. Rev, 5 (2018), 26–29. 30034911 10.1093/nsr/nwx137 PMC6051411

[28] Raghu VK, Ramsey JD, Morris A, Manatakis DV, Sprites P, Chrysanthis PK, , Comparison of strategies for scalable causal discovery of latent variable models from mixed data, Int. J. Data Sci. Anal, 6 (2018), 33–45. 30148202 10.1007/s41060-018-0104-3 PMC6096780

[29] Ramsey J, Glymour M, Sanchez-Romero R, Glymour C, A million variables and more: the fast greedy equivalence search algorithm for learning high-dimensional graphical causal models, with an application to functional magnetic resonance images, Int. J. Data Sci. Anal, 3 (2017), 121–129. 28393106 10.1007/s41060-016-0032-z PMC5380925

[30] Xing L, Guo M, Liu X, Wang C, Wang L, Zhang Y, An improved bayesian network method for reconstructing gene regulatory network based on candidate auto selection, BMC Genom, 18 (2017), 844. 10.1186/s12864-017-4228-y PMC5773867 29219084

[31] Zhang L, Rodrigues LO, Narain NR, Akmaev VR, bAIcis: A novel bayesian network structural learning algorithm and its comprehensive performance evaluation against open-source software, J. Comput. Biol, 27 (2020), 698–708. 31486672 10.1089/cmb.2019.0210 PMC7232674

[32] Andrews B, Ramsey J, Cooper GF, Scoring bayesian networks of mixed variables, Int. J. Data Sci. Anal, 6 (2018), 3–18. 30140730 10.1007/s41060-017-0085-7 PMC6101981

[33] Andrews B, Ramsey J, Cooper GF, Learning high-dimensional directed acyclic graphs with mixed data-types, Proc. Mach. Learn. Res, 104 (2019), 4–21. 31453569 PMC6709674

[34] Sedgewick AJ, Shi I, Donovan RM, Benos PV, Learning mixed graphical models with separate sparsity parameters and stability-based model selection, BMC Bioinform, 17 (2016), 175. 10.1186/s12859-016-1039-0 PMC4905606 27294886

[35] Jabbari F, Ramsey J, Spirtes P, Cooper G, Discovery of causal models that contain latent variables through bayesian scoring of independence constraints, Lect. Notes Comput. Sc, 10535 (2017), 142–157. 10.1007/978-3-319-71246-8_9 PMC5836552 29520396

[36] Ogarrio JM, Spirtes P, J R, A hybrid causal search algorithm for latent variable models, JMLR Workshop Conf. Proc, 52 (2016), 368–379. 28239434 PMC5325717

[37] Yu K, Liu L, Li J, Learning markov blankets from multiple interventional data sets, IEEE Trans. Neural Netw. Learn. Syst, 31 (2020). 10.1109/TNNLS.2019.2927636 31478874

[38] Chen J, Zhang R, Dong X, Lin L, Zhu Y, He J, , shinybn: an online application for interactive bayesian network inference and visualization, BMC Bioinform, 20 (2019), 711. 10.1186/s12859-019-3309-0 PMC6916222 31842743

[39] Eicher T, Patt A, Kautto E, Machiraju R, Mathé E, Zhang Y, Challenges in proteogenomics: a comparison of analysis methods with the case study of the dream proteogenomics sub-challenge, BMC Bioinform, 20 (2019), 669. 10.1186/s12859-019-3253-z PMC6923881 31861998

[40] Ramanan N, Natarajan S, Causal learning from predictive modeling for observational data, Front. Big Data, 3 (2020), 535976. 33693412 10.3389/fdata.2020.535976 PMC7931928

[41] Tasaki S, Sauerwine B, Hoff B, Toyoshiba H, Gaiteri C, Neto EC, Bayesian network reconstruction using systems genetics data: comparison of mcmc methods, Genetics, 199 (2015), 973–89. 25631319 10.1534/genetics.114.172619 PMC4391572

[42] Pratapa A, Jalihal AP, Law JN, Bharadwaj A, Murali TM, Benchmarking algorithms for gene regulatory network inference from single-cell transcriptomic data, Nat. Methods, 17 (2020). 10.1038/s41592-019-0690-6 PMC7098173 31907445

[43] Peters J, Mooij JM, Janzing D, Schölkopf B, Causal discovery with continuous additive noise models, J. Mach. Learn. Res, 15 (2014), 2009–2053,

[44] Kaur D, Sobiesk M, Patil S, Liu J, Bhagat P, Gupta A, , Application of bayesian networks to generate synthetic health data, J. Am. Med. Inform. Assoc, 28 (2020), 801–811. 10.1093/jamia/ocaa303 PMC7973486 33367620

[45] Young JB, Graham P, Penny R, Using bayesian networks to create synthetic data, Qual. Eng, 55 (2010), 363–366.

[46] Roozegar R, Soltani AR, On the asymptotic behavior of randomly weighted averages, Stat. Probabil. Lett, 96 (2015), 269–272.

